# A Prediction Model for Tacrolimus Daily Dose in Kidney Transplant Recipients With Machine Learning and Deep Learning Techniques

**DOI:** 10.3389/fmed.2022.813117

**Published:** 2022-05-27

**Authors:** Qiwen Zhang, Xueke Tian, Guang Chen, Ze Yu, Xiaojian Zhang, Jingli Lu, Jinyuan Zhang, Peile Wang, Xin Hao, Yining Huang, Zeyuan Wang, Fei Gao, Jing Yang

**Affiliations:** ^1^Department of Pharmacy, The First Affiliated Hospital of Zhengzhou University, Zhengzhou, China; ^2^Henan Key Laboratory of Precision Clinical Pharmacy, Zhengzhou University, Zhengzhou, China; ^3^Beijing Medicinovo Technology Co. Ltd, Beijing, China; ^4^Dalian Medicinovo Technology Co. Ltd, Dalian, China; ^5^McCormick School of Engineering, Northwestern University, Evanston, IL, United States

**Keywords:** prediction model, tacrolimus, daily dose, kidney transplant, machine learning, genetic polymorphism

## Abstract

Tacrolimus is a major immunosuppressor against post-transplant rejection in kidney transplant recipients. However, the narrow therapeutic index of tacrolimus and considerable variability among individuals are challenges for therapeutic outcomes. The aim of this study was to compare different machine learning and deep learning algorithms and establish individualized dose prediction models by using the best performing algorithm. Therefore, among the 10 commonly used algorithms we compared, the TabNet algorithm outperformed other algorithms with the highest R^2^ (0.824), the lowest prediction error [mean absolute error (MAE) 0.468, mean square error (MSE) 0.558, and root mean square error (RMSE) 0.745], and good performance of overestimated (5.29%) or underestimated dose percentage (8.52%). In the final prediction model, the last tacrolimus daily dose, the last tacrolimus therapeutic drug monitoring value, time after transplantation, hematocrit, serum creatinine, aspartate aminotransferase, weight, *CYP3A5*, body mass index, and uric acid were the most influential variables on tacrolimus daily dose. Our study provides a reference for the application of deep learning technique in tacrolimus dose estimation, and the TabNet model with desirable predictive performance is expected to be expanded and applied in future clinical practice.

## Introduction

Tacrolimus, a calcineurin inhibitor, is a widely acceptable used immunosuppressive drug in kidney transplant recipients (KTRs). The narrow therapeutic index of tacrolimus leads to a very close edge between therapeutic and toxic blood concentration (1). An inappropriate regimen of tacrolimus in the early phase after transplantation is associated with acute rejections if the therapeutic range is not achieved, or adverse events (nephrotoxicity, infections as well as the development of new-onset diabetes or hypertension) if the therapeutic range is exceeded (2–9). Therefore, even small dose adjustments should be carefully chosen, and an accurate estimation of the relationship between tacrolimus dose and dose-response is important for postoperative outcomes.

The predictable suitable dose of tacrolimus is critical for the health status of postoperative KTRs. The Kidney Disease: Improving Global Outcomes guidelines suggested that 5–15 ng/ml of tacrolimus trough levels should be used during the first 2–4 months post-transplantation, and reduced thereafter in KTRs to minimize toxicity and adverse effects (10). More effective immunosuppressive therapy protocol during the early phase after solid organ transplantation would not only enhance the quality and length of life but would also reduce the need for transplantation (11). How to translate technological innovations into a workable tacrolimus dosing tool for clinicians has been explored in recent studies.

A consensus report on therapeutic drug monitoring (TDM) of tacrolimus-personalized therapy was updated in 2019. The report considered the most relevant advances in tacrolimus pharmacokinetics, pharmacogenetics, pharmacodynamics, and immunologic biomarkers to assist professionals to individualize tacrolimus treatment (12). Clinicians usually made dosage adjustments based on TDM in the early post-transplantation phase, until it reached long-term maintenance immunosuppression levels. However, the dose required to achieve the targeted whole blood concentrations of tacrolimus varies among individuals (13, 14). Numerous factors have been reported that are associated with the expression of metabolic enzyme and transporter to affect the pharmacokinetics of tacrolimus, including patient demographics, gene polymorphisms, time after transplantation, concomitant medications, comorbidities, hematocrit, hepatic function, and so forth (15). Thus, how to synthesize these potential factors and translate them into the tacrolimus dose adjustment strategy for immunosuppressive therapy in KTRs is still necessary to be explored and confirmed.

To evaluate the relevance of individual factors to influencing tacrolimus effect, researchers attempted to develop clinically practicable dosing models for tacrolimus stable dose. Methods of multiple linear regression (MLR) and population pharmacokinetics (PPK) have been utilized in tacrolimus dosing models (12, 16–19). With the development of machine learning and deep learning techniques, more studies applied these techniques to enhance the model expression of the complicated relationship between individual factors and medication dose. Tang et al. first applied multiple machine learning algorithms to establish a stable dosing prediction model of tacrolimus in Chinese KTRs, and proved the method was superior to the MLR (20, 21). In addition to the algorithms used in Tang's research, some algorithms with more sophisticated principles were developed, such as eXtreme Gradient Boosting (XGBoost), light gradient boosting machine (LightGBM), Categorical Boosting (CatBoost), random forest (RF), and TabNet (a deep learning technique), which were highly recognized in the algorithm competitions (22–26). For dose prediction of continuous data, these algorithms based on the principle of decision trees and deep neural networks might be more suitable. Herein, we conducted this study to investigate the individualized factors that markedly affect tacrolimus stable dose as well as to identify the most workable algorithm for prediction of the dose regimen of Chinese KTRs.

Taking the tacrolimus dose prediction model in this study as an example, with the increasing data amount of input subjects, the machine learning model could constantly optimize the parameters to achieve better accuracy and practicality. It is prospective to revolutionize the prescription and promote individualized medication in the future.

## Methods

### Study Population

**Figure 1:**
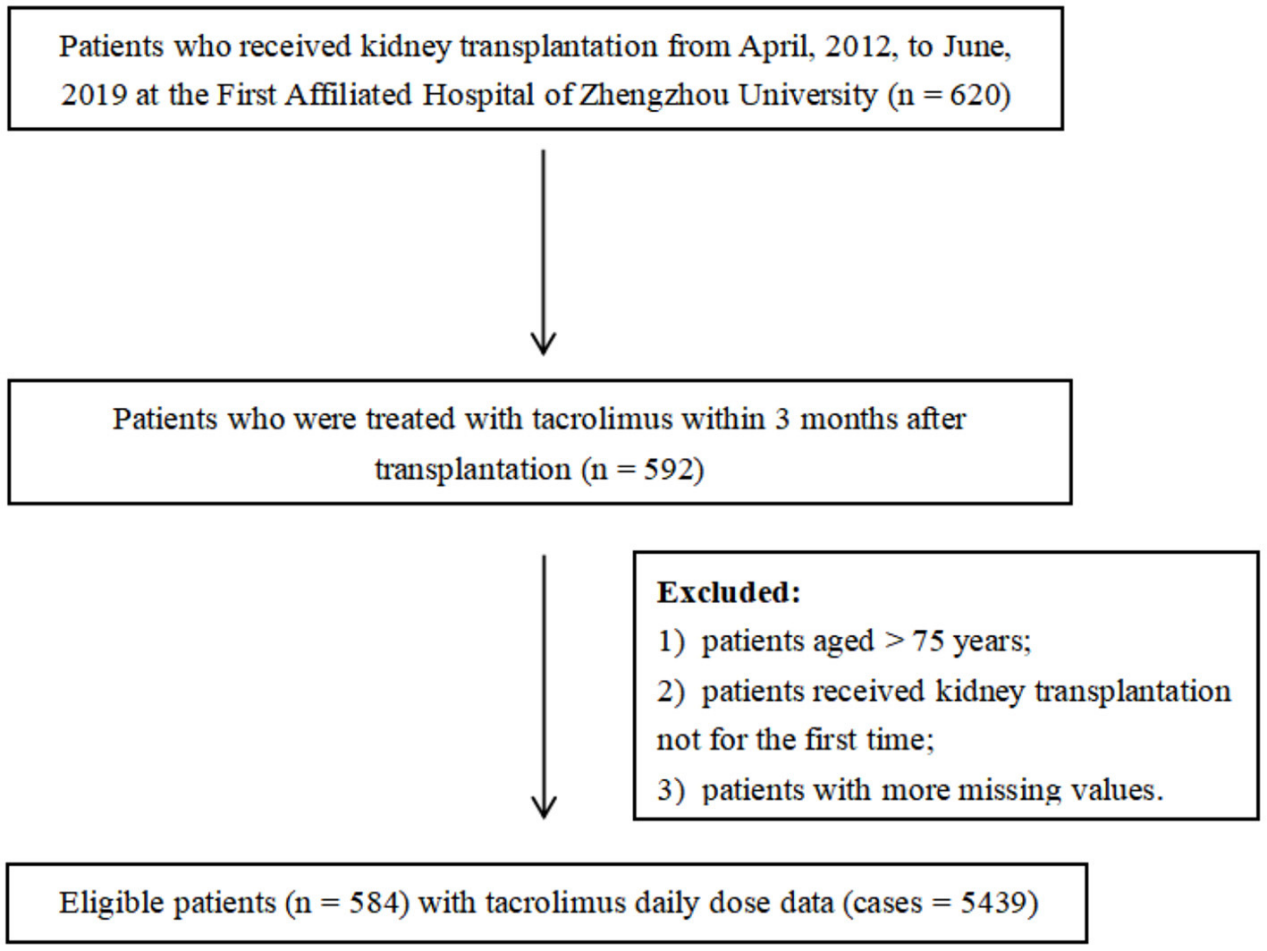
Enrollment of patients.

A retrospective analysis of recipients who received kidney allografts between April 2012 and June 2019 was performed. Clinical and demographic data were collected from the medical records in the First Affiliated Hospital of Zhengzhou University, China. The study protocol was approved by the Ethics Committee of the First Affiliated Hospital of Zhengzhou University (2020-KY-147). Eligible for enrollment were patients aged younger than 75 years and were treated with tacrolimus as part of their immune-suppressive regimen within 3 months post-transplantation. All recipients went through kidney transplantation for the first time and the donors were relatives and non-relatives. Patients who did not meet the criteria aforementioned and those with more missing values (missing rate over 20%) were excluded. Some variables showing the remarkable influence on tacrolimus dose even with a high percentage of missing values were not deleted and were interpolated subsequently (such as CYP3A5 and ABCB1). In the end, 584 patients with 5,439 cases of tacrolimus daily dose were included. The enrollment of patients is displayed in Figure 1.

### Data Collection and Processing

**Figure 2:**
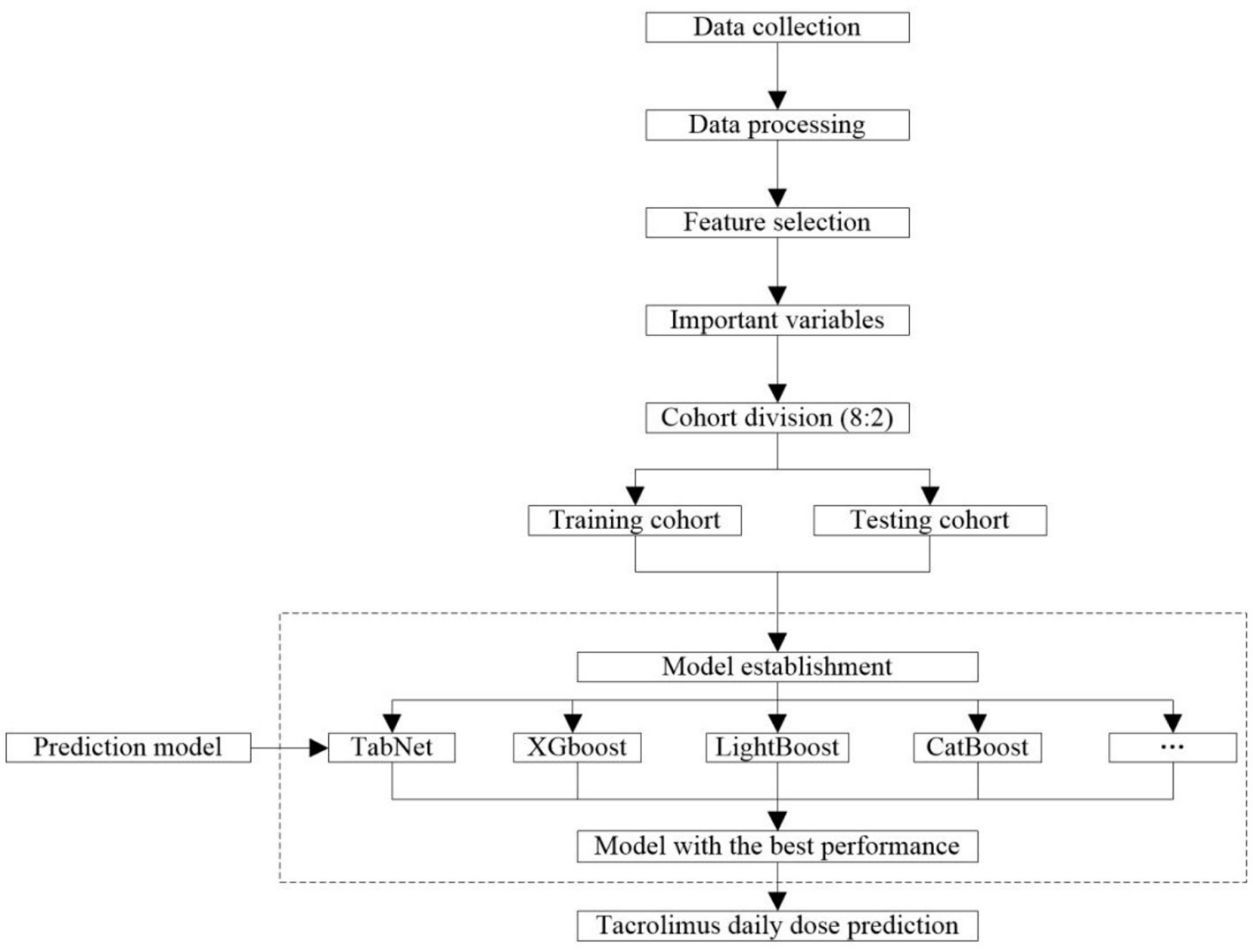
Workflow of data processing and modeling.

The workflow of collecting and processing data is shown in Figure 2. Data of laboratory tests, medication, and diagnosis were collected from the patients who met the criteria. We collected the same type of drug combinations, including glucocorticoid (GC), calcium channel blockers (CCB), PPI (proton pump inhibitor), Wuzhi softgel, enzyme inducer, and mycophenolic acid (MPA) (Supplementary Table S1). The pathological status included a diagnosis of diarrhea, abdominal pain, abdominal distension, belching, anorexia, and gastrointestinal motility recovery. The daily tacrolimus dose equaled the sum of the doses prescribed by each order on that day. All results of successive TDM tests were retained because the order of tacrolimus that was closest to these tests was a long-term order, which considered that the corresponding medication conditions of these TDM tests were the same. For initial medication cases, days after transplantation = time of tacrolimus administration - time of kidney transplantation. For adjusting medication cases, days after transplantation = time of the last TDM test - time of kidney transplantation. The laboratory data were taken from the closest test results before the first administration of tacrolimus after kidney transplant surgery. The concomitant medication data were taken from results of 3 days before the first administration of tacrolimus to the time before the first TDM test.

### Variables Selection

A univariate correlation testing procedure was implemented to remove all variables that were not directly related to tacrolimus daily dose. The Pearson Correlation test was applied for continuous variables by investigating the association between the variable and outcome. In addition, the Mann–Whitney U test was used for binary variables, and Analysis of Variance (ANOVA) was used for multiclass variables. This screening procedure adopted the criteria of *p*-value ≤ 0.05 to recruit impact variables in the following stages. Subsequently, the univariates were further screened by machine learning, and their importance scores were calculated and ranked. Those ranked at the top of the list were selected as the important variables to construct the model.

Before the machine learning algorithms, RF was applied to interpolate missing data (27). The missing values were then imputed using multiple regression sequentially based on different types of missing covariates. After imputation of RF, the remaining data set was used for the modeling stage, and the missing percentages after interpolation are summarized in Supplementary Table S2.

### Model Construction

The workflow of modeling is shown in Figure 2. A total of 10 frequently used algorithms including deep learning method and machine learning methods were selected for generating the predictive models using the processed feature set. These algorithms were XGBoost, LightGBM, CatBoost, Gradient Boosted Decision Tree (GBDT), RF, support vector regression (SVR), K-nearest neighbor (KNN), Least Absolute Shrinkage and Selection Operator (LASSO) regression, ridge regression (RR), linear regression, and TabNet (22–26, 28–34). The data set was randomly allocated to the training cohort used to train the model and the testing cohort used to verify the model according to 8:2 ratio. The predictive performance of these algorithms is compared in this study. To minimize overfitting and obtain reliable results, 5-fold cross-validation was used to refine model prediction performance. The final model was constructed using the algorithm with the best predictive capacity based on the selected important variables.

### Model Evaluation

To evaluate and compare the predictability of models, we primarily assessed the R-square (R^2^), mean absolute error (MAE), mean square error (MSE), root mean square error (RMSE), and percentage of overestimated or underestimated dose in the testing cohort. More specifically, R^2^ is the squared correlation between predicted and actual dose value (square root of tacrolimus dose). The MAE is the average of the absolute difference between the actual and the predicted dose values in this case. The RMSE is the square root of the mean square error between the predicted dose and actual dose, and MSE is the average value of the distance between each predicted dose and the average actual dose.

## Results

### Baseline Information

Based on the whole data set, a total of 584 KTRs with 5,439 cases of dose data were identified in this study. The baseline information of the study population is shown in Table 1, including information regarding tacrolimus treatments, patient demographic information, combinations, genetic polymorphism, assay index, kidney transplantation information, and some other body conditions. The continuous variables were described by “median (interquartile range, IQR)”, and the classification variables were described by “frequency (%)”. The median age of patients in this study was 32 (IQR 26–41), and the proportion of female patients was 26.8%. In terms of drug combinations, nifedipine occupied the highest proportion of CCB (28.46 %), a median daily dose of GC was 18.8 (IQR 12.0–25.0) mg, and PPI (98.31 %), Wuzhi softgel (90.5 %), and MPA (71.8 %) had very high proportion in the study population.

**Table 1:** Baseline characteristic of study population.

**Variables**	**Total cohort** **(Cases = 5,439)**	**Missing rate (%)**
Age (yrs), median (IQR)	32 (26–41)	2.89
Sex, *n* (%)		3.44
Female	1456 (26.8%)	
Male	3796 (69.8%)	
Height (cm), median (IQR)	170 (160–173)	3.05
Weight (kg), median (IQR)	59 (50–65)	3.05
BMI (kg/m^2^), median (IQR)	20.6 (18.7–22.4)	3.05
Tacrolimus dose (mg/d), median (IQR)	5.0 (3.5–6.0)	0
Last tacrolimus dose (mg/d), median (IQR)	4.5 (3.5–6.0)	0
Last tacrolimus TDM (ng/mL), median (IQR)	9.5 (7.5–12.0)	0
GC dose (mg/d), median (IQR)	18.8 (12.0–25.0)	0
Nifedipine, *n* (%)	1,548 (28.46%)	0
Levamlodipine besylate, *n* (%)	868 (15.96%)	0
Valsartan amlodipine, *n* (%)	1,413 (25.98%)	0
Nikadine, *n* (%)	147 (2.70%)	0
Felodipine, *n* (%)	367 (6.75%)	0
PPI, *n* (%)	5,347 (98.31%)	0
Wuzhi softgel, *n* (%)	5,065 (90.5%)	0
Enzyme inducer, *n* (%)	11 (0.2%)	0
MPA, *n* (%)	3,905 (71.8%)	0
CYP3A5*3, n (%)		52.07
A/A	231 (4.2%)	
A/G	1,161 (21.3%)	
G/G	1,215 (22.3%)	
*ABCB1, n* (%)		52.07
C/C	1,016 (18.7%)	
C/T	1,129 (20.8%)	
T/T	462 (8.5%)	
UA (umol/L), median (IQR)	343 (261–429)	3.92
SCr (umol/L), median (IQR)	262 (145–571)	3.92
AST (U/L), median (IQR)	13 (10–18)	4.04
TBIL (umol/L), median (IQR)	8.2 (5.9–11.7)	4.03
RBC (10^12^/L), median (IQR)	3.27 (2.87–3.76)	1.62
Hb (g/L), median (IQR)	99 (87.9–111)	1.47
HCT (L/L), median (IQR)	0.30 (0.27–0.35)	1.47
NEU (%), median (IQR)	89.1 (76.6–95.3)	1.47
LYM (%), median (IQR)	4.9 (1.7–13.7)	1.47
Time after transplantatio*n* (d), median (IQR)	10 (5–20)	0
Living relative's organ transplantation, *n* (%)	788 (14.5%)	0
Hypertension, *n* (%)	3,783 (69.6%)	0
Diabetes, *n* (%)	23 (0.4%)	0
Other pathological status, *n* (%)	515 (9.5%)	0

*BMI, body mass index; GC, glucocorticoid; PPI, proton pump inhibitor; MPA, mycophenolic acid; UA, uric acid; SCr, serum creatinine; AST, aspartate aminotransferase; TBIL, total bilirubin; RBC, red blood cells; Hb, hemoglobin; HCT, hematocrit; NEU, neutrophil; LYM, lymphocyte*.

### Univariate Analysis

We determined the significant associations between univariate and tacrolimus daily dose (*p*-value ≤ 0.05). Through this screening procedure, 25 variables were found significant, and entered the subsequent processing and modeling stages (Table 2).

**Table 2:** Univariate analysis results.

**Variable**	**Statistic (r/U/F)**	***p*–value**
Last tacrolimus TDM	−0.146	<0.001
Last tacrolimus daily dose	0.542	<0.001
Time after transplantation	−0.352	<0.001
Age	0.143	<0.001
Sex	1870835.5	<0.001
Height	0.397	<0.001
Weight	0.406	<0.001
BMI	0.296	<0.001
GC dose	0.127	<0.001
PPI	1,91,491	<0.001
Wuzhi softgel	7,06,284	<0.001
Levamlodipine besylate	19,64,818	0.651
Valsartan amlodipine	2789975.5	0.28
Nifedipine	2908343.5	0.046
Nikadine	4,48,043	0.002
Felodipine	857026	0.01
Enzyme inducer	27028.5	0.584
MPA	2568291.5	<0.001
CYP3A5*3	359811.5	<0.001
*ABCB1*	1.464	0.232
UA	−0.065	<0.001
SCr	0.180	<0.001
AST	−0.110	<0.001
TBIL	−0.027	0.05
RBC	0.020	0.142
Hb	−0.020	0.146
HCT	0.051	<0.001
NEU%	0.182	<0.001
LYM%	−0.171	<0.001
Hypertension	28,87,309	<0.001
Diabetes	51,447	0.146
Living donor kidney transplantation from relatives	15,86,384	<0.001
Pathological status	11,80,315	0.009

*BMI, body mass index; GC, glucocorticoid; PPI, proton pump inhibitor; MPA, mycophenolic acid; UA, uric acid; SCr, serum creatinine; AST, aspartate aminotransferase; TBIL, total bilirubin; RBC, red blood cells; Hb, hemoglobin; HCT, hematocrit; NEU, neutrophil; LYM, lymphocyte*.

### Comparison of Predictive Algorithms

**Figure 3:**
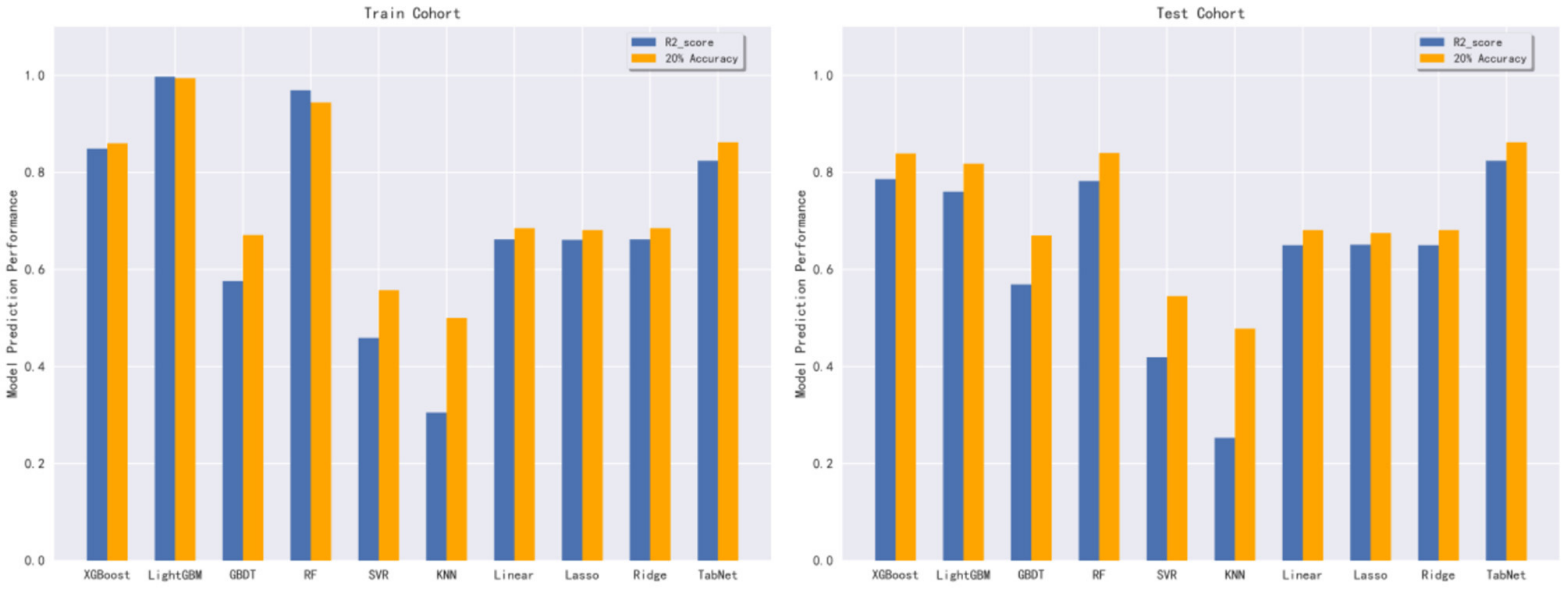
Model prediction performance after the 5-fold cross-validation.

**Figure 4:**
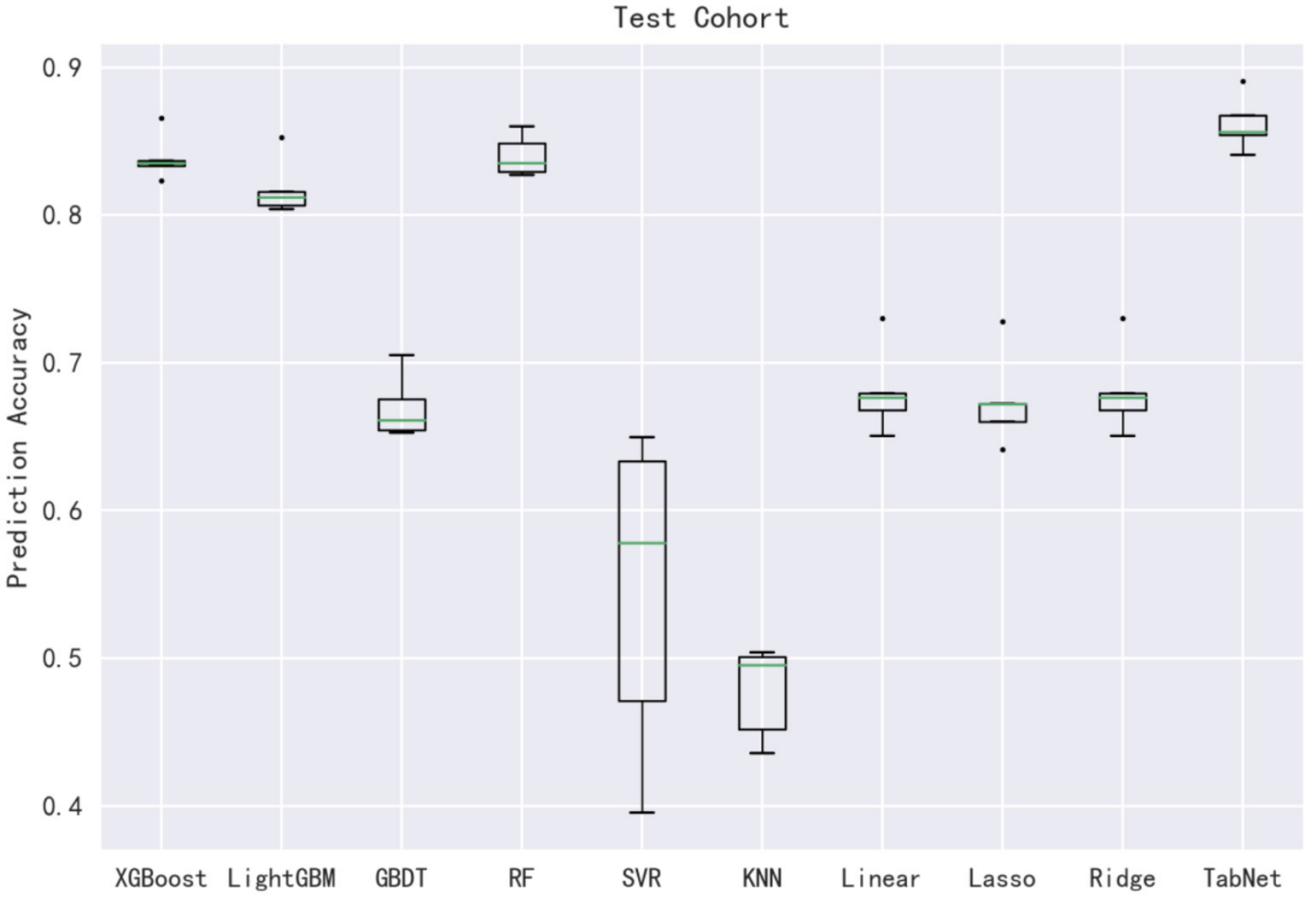
Prediction accuracy of 10 models.

In this subsection, 10 machine learning methods were compared in terms of R^2^, MAE, MSE, and RMSE in the testing cohort. A large R^2^ indicated that the established model had a good fitting, and small errors (i.e., MAE, MSE, and RMSE) indicated higher predictive accuracy overall. Strikingly, the TabNet algorithm outperformed all other methods in R^2^ (0.813), MAE (0.464 mg/d), MSE (0.615 mg/d), and RMSE (0.784 mg/d). The percentage of the overestimated dose (5.29 %) ranked in the top 4 and the percentage of underestimated dose (8.52 %) in the testing cohort of TabNet ranked in the top 1, demonstrating a robust predictive performance. The specific results of the model evaluation are listed in Table 3. The model prediction performance after the 5-fold cross-validation is displayed in Figure 3. It could be seen that after 5-fold cross-validation, TabNet had the highest R^2^ and 20% accuracy value in the testing cohort, indicating its best fitness and accuracy among 10 algorithms. Regarding the prediction accuracy of 10 models, a boxplot is illustrated in Figure 4, indicating that TabNet had the highest prediction accuracy among 10 models.

**Table 3:** R^2^, MAE, MSE, RMSE results, and percentage of overestimated or underestimated dose in the testing cohort of each predictive algorithms.

**Predictive algorithm**	**R^**2**^**	**MAE (mg/d)**	**MSE (mg/d)**	**RMSE (mg/d)**	**Underestimated dose (%)**	**Overestimated** **dose (%)**
XGBoost	0.786	0.488	0.681	0.821	4.60	11.51
LightGBM	0.760	0.523	0.761	0.868	7.21	10.97
GBDT	0.569	0.923	1.368	1.169	7.75	25.28
RF	0.782	0.461	0.693	0.829	4.91	11.09
SVR	0.419	1.010	1.829	1.343	31.34	14.12
KNN	0.253	1.241	2.365	1.537	20.56	31.68
Linear regression	0.650	0.756	1.110	1.050	12.28	19.64
LASSO regression	0.651	0.756	1.108	1.050	12.50	20.02
Ridge regression	0.650	0.756	1.110	1.050	12.28	19.64
TabNet	0.824	0.468	0.558	0.745	5.29	8.52

*GBDT, Gradient Boosted Decision Tree; RF, random forest; SVR, support vector regression; KNN, K-nearest neighbor; LASSO, Least Absolute Shrinkage and Selection Operator*.

### Dose Prediction Model Using TabNet

Based on the comparison of algorithms, the TabNet model was verified as the best model for predicting the dose of tacrolimus in KTRs. As shown in Table 4, the accuracy of the predicted dose within ±20, ±30, and ±40% of the actual dose was 86.19, 91.33, and 93.48%, respectively. It automatically recruited the most influential variables referring to the sum of their importance scores. Table 5 lists the top 10 important variables to predict tacrolimus dose and their importance scores in the TabNet model, including the last tacrolimus daily dose (0.316), the last tacrolimus TDM (0.219), time after transplantation (0.083), hematocrit (HCT; 0.079), SCr (0.068), aspartate aminotransferase (AST; 0.058), weight (0.037), *CYP3A5* (0.037), BMI (0.036), and uric acid (UA; 0.021).

**Table 4:** The prediction accuracy of the predicted value in different confidence intervals.

**Model**	**Accuracy of the predicted dose within ±20% of the actual dose**	**Accuracy of the predicted dose within ±30% of the actual dose**	**Accuracy of the predicted dose within ±40% of the actual dose**
XGBoost	83.89%	89.37%	92.41%
LightGBM	81.82%	88.61%	91.87%
GBDT	66.97%	75.87%	81.24%
RF	84.01%	89.49%	92.36%
SVR	54.54%	71.11%	83.35%
KNN	47.76%	64.87%	75.18%
Linear regression	68.08%	80.51%	86.07%
Lasso regression	67.47%	80.59%	86.03%
Ridge regression	68.08%	80.51%	86.07%
TabNet	86.19%	91.33%	93.48%

**Table 5:** Ranking of importance scores.

**Variable**	**Importance score**
Last tacrolimus daily dose	0.316
Last tacrolimus TDM	0.219
Time after transplantation	0.083
HCT	0.079
SCr	0.068
AST	0.058
Weight	0.037
*CYP3A5*	0.037
BMI	0.036
UA	0.021

*HCT, hematocrit; SCr, serum creatinine; AST, aspartate aminotransferase; BMI, body mass index; UA, uric acid*.

## Discussion

Tacrolimus dose adjustment is generally reactive administration, which has a wide variability in the early phase after transplantation (10, 35). Our research was designed based on the data of appropriate tacrolimus administration among Chinese, which comprehensively analyzed the widely varying multivariate and provided an accurately predicted dose during the early postoperative period. Overall, we found different performances of the 10 algorithms that were used to predict tacrolimus dosing in the Chinese KTRs. When all the cases were investigated, the TabNet algorithm achieved the best performance.

As a novel deep learning technique, whether it is unsupervised learning for filling missing features or supervised learning for actual decision-making, the TabNet encoder is used to encode the input features first; then the missing features are filled separately with decoder connections according to different uses, or connected with the full connection layer to achieve the final decision. The TabNet encoder architecture is mainly composed of a feature transformer, an attentive transformer, and feature masking. The decoder uses the feature transformer layer to reconstruct the encoded features to the raw data table features (26). Moreover, TabNet uses a sequential attention mechanism to choose a subset of meaningful features to process at each decision step, enabling interpretability and more efficient learning as the learning capacity used for the most salient features. TabNet employs a single deep learning architecture for feature selection and reasoning (26). Additionally, based on retaining the end-to-end and representation learning characteristics of deep neural networks, TabNet also has the advantages of tree model interpretability and sparse feature selection (36). Other studies based on real-world data showed that TabNet outperformed ensemble tree-based algorithms since it could process highly non-linear relationships with its depth, without overfitting due to instance-wise feature selection (26). However, there are some limitations of implementing or translating TabNet compared to other machine learning algorithms, including (i) TabNet runs slowly, consumes more time and resources, and even runs several times longer than XGBoost; (ii) TabNet could not give full play to its advantages when the amount of data was not very large.

Furthermore, compared with conventional modeling methods, machine learning and deep learning techniques have indubitable advantages in dealing with real-world data, such as (i) machine learning and deep learning can deal with more complex, high-dimensional, and interactive variables from the clinical environment, which is lacking in conventional models; (ii) machine learning and deep learning models have a stronger generalization and better accuracy than conventional models (37–39). Recently, the application of machine learning and deep learning techniques on individualized dose prediction models has been approbatory, such as a novel vancomycin dose prediction model through XGBoost and warfarin maintenance dose prediction through LightGBM (40, 41). With the increasing number of input subject data, machine learning and deep learning models can continually optimize parameters to achieve better performance and practicality. Herein, we calculated and compared model predictive performance among all algorithms. The R^2^ of the testing cohort of our tacrolimus dose prediction model using TabNet reached 0.824 in the overall comparison. Compared to the results of Tang et al., which applied eight machine learning algorithms, including multiple linear regression, artificial neural network, regression tree, multivariate adaptive regression splines, boosted regression tree, SVR, RF, LASSO regression, and Bayesian additive regression trees (RF achieved the lowest MAE of 0.73 in the validation cohort), our prediction model could achieve a better outcome (TabNet achieved the MAE of 0.464) (20).

After estimating the importance of variables through TabNet, the last tacrolimus daily dose, the last tacrolimus TDM, time after transplantation, HCT, SCr, AST, weight, *CYP3A5*, BMI, and UA were identified as the most influential factors in dose prediction. In the previous PPK models, among the transplant recipients, the factors most associated with whole blood apparent clearance variation for tacrolimus included cytochrome *CYP3A5*, weight, HCT, postoperative days, and hepatic function (e.g., AST), SCr, and the *CYP3A5* polymorphism was the most frequently included variable (12, 17–19, 42). In our deduction, the important variables were consistent with the result of previous studies. Due to the extensibility of the machine learning model, the model could be further improved after the addition of learnable data and more unrecognized novel influencing variables could be mined. Additionally, the machine learning model had more flexibility in that the recommended dose varies with the updated influencing variables, thus it was useful for the next medication prediction. Nevertheless, the PPK model generally just predicts the initial medication. Third, according to the second consensus report about TDM of tacrolimus-personalized therapy issued by the Immunosuppressive Drugs Scientific Committee of the International Association of Therapeutic Drug Monitoring and Clinical Toxicity in 2019, the range of R^2^ was unstable varying from 0.27 to 0.99 in the PPK models, whereas our model had a good performance of R^2^ = 0.813 (12).

The last tacrolimus daily dose and TDM value were closely related to the prediction of the next tacrolimus dose, and the time after transplantation had been chosen which was beneficial for considering relevant factors that change with time (19). In this study, the height, weight, and gender of patients were regarded as control factors, since we considered the integrity of their data in our study population and their fixed effects. Above all, the physiology existed a wide discrepancy between obese and non-obese individuals (43). Multiple studies verified in humans and rodents, that obesity changes the mRNA or protein expression levels of hepatic CYP3A in the liver and intestine, which was a major oxidatively metabolizing enzyme of tacrolimus (43–45). Sawamoto et al. reported that obese patients could maintain tacrolimus concentration well at lower doses compared with non-obese patients (46). Hence, weight and BMI were important in predicting tacrolimus dose. Patients expressing at least one CYP3A5^*^1 allele need a higher tacrolimus dose than those not carrying this allele (CYP3A5^*^3/^*^3) to reach the same blood concentration (12).

As mentioned above, tacrolimus is mainly metabolized in the liver and intestinal wall through isoforms of CYP3A enzymes (CYP3A4 and CYP3A5). The major enzyme involved in its biological transformation is CYP3A5, and the catalytic efficiency of CYP3A4 is lower (41). The association between CYP3A5 and tacrolimus dose has been observed in KTRs (16, 18). Asberg A et al. found that the *CYP3A5* genotype led to higher clearance and lower bioavailability of tacrolimus, and its inclusion in the model could improve dose prediction (16). The marked pharmacokinetic variability of tacrolimus was partly due to the *CYP3A5* genotype, therefore, CYP3A5 was important for our predictive results. According to the consensus report, the *ABCB1* rs1045642 (3435C>T) was reported to affect the transporter activity which could influence the concentration of tacrolimus (12). However, this factor was excluded due to the univariate analysis results in this research.

In terms of biochemical parameters affecting the tacrolimus dose, a PPK model proved that lower SCr and lower HCT levels were identified as important factors to enhance tacrolimus clearance (36). The remarkable impact of SCr on tacrolimus dose herein was consistent with the results of previous research, which illustrated that decreased SCr has a significant association with tacrolimus dose (47). Furthermore, HCT explained 4~14% of the variability in tacrolimus dose requirements and clearance *in vivo* (48). A high percentage of tacrolimus in blood was binding to erythrocytes, which could be influenced by HCT and red blood cell count significantly (49). Multiple studies point out that HCT varied hugely in transplant recipients and increased widely after transplantation, and the concentration of tacrolimus associated with erythrocytes tended to increase along with the augment of HCT in post-transplant patients (49, 50). Additionally, UA, as an indicator of kidney function, was demonstrated to be increased after tacrolimus therapy in kidney or liver transplant recipients (51, 52). The increasing UA level may imply a decline in kidney function, which may need a change in the tacrolimus regimen, hence, UA could be regarded as a posterior factor that affects the tacrolimus dose.

Additionally, drug–drug interactions with tacrolimus dose-response were also considered in the study. We included several kinds of concomitant medications, such as GC, CCB, PPI, Wuzhi softgel, enzyme inducer, and MPA. Of which, GC, Wuzhi softgel, and MPA were administered in the majority of KTRs, and unbalanced between positive and negative samples, hence, there was no significant difference among the participants. However, based on clinical experience, these drugs could influence tacrolimus concentration. For instance, according to Yan et al. and Wang et al., Wuzhi softgel could influence tacrolimus elimination and noticeably increase its drug concentration in Chinese organ transplant patients (53, 54). The effect of these concomitant drugs on tacrolimus might be further investigated based on balanced clinical data in the future.

In conclusion, this study was rigorously designed to mine in-depth the clinical and genetic influencing factors in KTRs treated with tacrolimus after kidney transplantation. In our study, a deep learning technique, TabNet, was utilized based on the multidimensional database to select the important factors relating to tacrolimus dose, which contributed to the practicable prediction accuracy of tacrolimus dosage in KTRs. The application of our proposed model for estimating the appropriate dose of tacrolimus provides recommendations for a clinically required therapeutic regimen.

## Data Availability Statement

The original contributions presented in the study are included in the article/Supplementary Material, further inquiries can be directed to the corresponding authors.

## Ethics Statement

Written informed consent was obtained from the individual(s) for the publication of any potentially identifiable images or data included in this article.

## Author Contributions

QZ and JY designed the study. ZY and FG provided medical support. XZ and JL guided the research. GC extracted the data. XT and PW collected and assembled the data. JZ, YH, and ZW analyzed the data. XH wrote the manuscript. All authors read and approved the final manuscript for publishing.

## Funding

This work was supported by the National Key R&D Program of China (Grant No. 2020YFC2008304), the Youth Program of National Natural Science Foundation of China (No. 81903704).

## Conflict of Interest

ZY, JZ, ZW, and FG are employed by Beijing Medicinovo Technology Co. Ltd, Beijing, China. XH is employed by Dalian Medicinovo Technology Co. Ltd, Dalian, China. The remaining authors declare that the research was conducted in the absence of any commercial or financial relationships that could be construed as a potential conflict of interest.

## Publisher's Note

All claims expressed in this article are solely those of the authors and do not necessarily represent those of their affiliated organizations, or those of the publisher, the editors and the reviewers. Any product that may be evaluated in this article, or claim that may be made by its manufacturer, is not guaranteed or endorsed by the publisher.
