# Supplementary material for: A Prediction Model for Tacrolimus Daily Dose in Kidney Transplant Recipients With Machine Learning and Deep Learning Techniques
Source: Front Med (Lausanne). 2022 May 27;9:813117. doi: 10.3389/fmed.2022.813117 (PMC9197124; doi:10.3389/fmed.2022.813117)
Supplement: Supplementary file 1 [file Table_1.DOCX]

**Supplementary Materials**

**Supplementary TABLE S1.** Included combination medications in candidate variables.

| **Drug type** | **Contained drugs** |
| --- | --- |
| GC | Methylprednisolone or/and prednisone, convert the daily dose of prednisolone into the equivalent dose of methylprednisolone (*1.25) |
| CCB | Levamlodipine besylate, valsartan amlodipine, nifedipine, nikadine, felodipine. |
| PPI | Pantoprazole, telazol, lansoprazole. |
| Enzyme inducer | St. John's wort (hypericum perforatum), barbiturates, phenytoin sodium, rifampin, carbamazepine. |
| MPA | Mycophenolate mofetil. |

Abbreviation: GC, glucocorticoid; CCB, calcium channel blockers; PPI, proton pump inhibitor, MPA, mycophenolic acid.

**Supplementary TABLE S2**. Missing rate of each variable after interpolation.

| **No.** | **Variable** | **Missing rate (%)** | **No.** | **Variable** | **Missing rate (%)** |
| --- | --- | --- | --- | --- | --- |
| 1 | Last tacrolimus TDM | 0 | 14 | Nikadine | 0 |
| 2 | Last tacrolimus dose | 0 | 15 | Felodipine | 0 |
| 3 | Time after transplantation | 0 | 16 | MPA | 0 |
| 4 | Age | 0 | 17 | *CYP3A5*3* | 0 |
| 5 | Sex | 0 | 18 | UA | 4.18 |
| 6 | Height | 0 | 19 | SCr | 4.18 |
| 7 | Weight | 0 | 20 | AST | 4.10 |
| 8 | BMI | 0 | 21 | HCT | 1.69 |
| 9 | GC dose | 0 | 22 | NEU% | 1.69 |
| 10 | Latrazole | 0 | 23 | LYM% | 1.69 |
| 11 | Wuzhi softgel | 0 | 24 | Hypertension | 0 |
| 12 | PPI | 0 | 25 | Living donor kidney transplantation from relatives | 0 |
| 13 | Nifedipine | 0 | 26 | Pathological status | 0 |

Abbreviation: BMI, body mass index; GC, glucocorticoid; PPI, proton pump inhibitor; MPA, mycophenolic acid; UA, uric acid; SCr, [serum](file:///C:/Users/ndyx/AppData/Local/youdao/dict/Application/8.9.3.0/resultui/html/index.html#/javascript:;) [creatinine](file:///C:/Users/ndyx/AppData/Local/youdao/dict/Application/8.9.3.0/resultui/html/index.html#/javascript:;); AST, aspartate aminotransferase; HCT, hematocrit; NEU, neutrophil; LYM, lymphocyte.
